# Optimization of citrulline production from a *Bacillus subtilis* BH-01 isolated from raw buffalo milk

**DOI:** 10.1186/s12866-025-03768-0

**Published:** 2025-02-10

**Authors:** Marwa A. K. Mansour, Salah G. Ali, Manal A. M. Hassan, Fify A. Gabra, Asmaa M. M. Mawad

**Affiliations:** 1 Botany and Microbiology Department, Faculty of Science, Al-Azhar University, Assiut, 71524 Egypt; 2 Botany and Microbiology Department, Faculty of Science, Assiut University, Assiut, 71516 Egypt; 3 Food Science and Technology Department, Faculty of Agriculture, Assiut University, Assiut, 71526 Egypt; 4 Metabolic and Genetics Disorder Unit, Faculty of Medicine, Assiut University, Assiut, 71526 Egypt; 5 Department of Biology, College of Science, Taibah University, Madinah, Saudi Arabia

**Keywords:** Arginine, *Bacillus*, Box-Behnken design, Citrulline, Probiotic

## Abstract

The main purpose of this study was to optimize the L-citrulline production process using Plackett-Burman and Box-Behnken designs. L-citrulline-producing bacterium BH-01 was isolated from raw buffalo milk. The isolate was tested for probiotic activities such as tolerance to simulated gastric and intestinal juices, antagonistic activity against six antibiotic-resistant bacteria, and temperature tolerance. L-citrulline production and arginine deiminase (ADI) activity were optimized using statistical designs. The bacterial isolate was molecularly identified as *Bacillus subtilis* strain AUMC B-498 (accession number PP574248.1). The strain exhibited resistance at pH 2.0 and bile salt 0.5% for a two-hour exposure period. It could inhibit the growth of *Escherichia coli*, *Klebsiella pneumonia*, *Serratia* sp., *Staphylococcus aureus*, methicillin*-*resistant *Staphylococcus aureus* (MRSA), and *Streptococcus pneumoniae.* From the results of statistical optimization, the Plackett-Burman design identified temperature, L-arginine, incubation period, and peptone as the most effective factors among the eight selected variables. Based on these, the Box-Behnken design was used to optimize the factors required to maximize citrulline production. The maximum L-citrulline was 632.5 µg/L, and ADI activity was 1.42 U/mL. Therefore, BH-01 isolated from Buffalo milk might be a promising candidate in food, biotechnological, and pharmaceutical applications due to its dual functionality for citrulline production and probiotic characteristics.

**Supplementary Information:**

The online version contains supplementary material available at 10.1186/s12866-025-03768-0.

## Introduction

L-citrulline is a non-proteinogenic amino acid that actively participates in various metabolic pathways, significantly impacting physiological processes [1]. Microbial citrullination refers to the process by which certain microorganisms convert arginine residues in proteins or peptides into citrulline via microbial enzymes [2]. While microbial citrullination is more commonly studied in a biological or medical context [2, 3], it also has potential applications in industry because of its unique ability to alter protein structure and function. In pharmaceuticals, L-Citrulline can serve as a precursor for developing therapies targeting cardiovascular diseases or enhancing nitric oxide production [4–6]. Citrullination pathways, employed in the production of L-form amino acids, are essential for synthesis of various antibiotics. In food technology, citrulline provides functional benefits, such as improving protein solubility and digestibility [7]. In the textile industry, citrullination is used to enhance the softness or flexibility of leather products by modifying proteins within the hide [8]. These innovations can lead to the development of novel products, such as functional beverages, personalized supplements, and advanced drug formulations [9].

Many approaches have been applied for the production of citrulline, such as chemical synthesis [10], natural extraction from plants [11, 12], and microbial fermentation and enzymatic synthesis [13–15]. The method of enzymatic synthesis and microbial fermentation is a preferable because of substrate availability, “in particular” L-arginine, high production yield, simple operation process and purification steps [16]. L-arginine serves as the starting material or reactant in the enzymatic method for L-citrulline production. ADI (L-Arginine Deiminase, E.C. 3.5.3.6) is an enzyme that catalyzes the irreversible hydrolysis of the imino group of L-arginine to L-citrulline, and ammonia [17, 18]. Optimizing these enzymatic steps through genetic and process engineering forms the foundation for improving citrulline production [19]. Process parameters such as pH, temperature, and oxygen levels are critical for maximizing the microbial activity and citrulline yield [20]. Maintaining an optimal pH (typically 6.5–7.5) enhances enzyme activity and microbial growth. Identifying the ideal temperature (usually 30–37 °C) for the selected strain ensures an efficient metabolism [14]. The composition of the fermentation medium significantly influences citrulline production. Glucose, sucrose, and glycerol are commonly used as primary carbon sources. Ammonium salts or organic nitrogen sources, such as peptone, support microbial growth and citrulline biosynthesis [14]. In addition to the mathematical models simulate fermentation dynamics, optimizing nutrient feed rates and timing for maximum productivity [21]. Fed-batch fermentation, where nutrients are added incrementally, prevents substrate inhibition and extends the production phase [14].

Specific microorganisms, either naturally occurring or genetically engineered, are employed for their ability to synthesize L-citrulline through metabolic pathways such as bacterial species belonging to the genera *Bacillus*, *Pseudomonas*, *Lactobacillus*, and *Clostridium via* induction of ADI [4, 20, 22, 23]. Selection of probiotic strains as candidates for L-citrulline production is promising due to their ability to metabolize substrates effectively and their proven safety for use in food, pharmaceutical, and nutraceutical industries [24]. Most probiotics, such as lactic acid bacteria possess enzymatic pathways (e.g., the arginine deiminase pathway) that can convert arginine to citrulline, making them efficient natural producers [25, 26]. *Bacillus subtilis* was recently added by the European Food Safety Authority (EFSA) to the qualified presumption of safety list of biological agents and allowing their use in food industries [27].

Statistical optimization techniques are essential in biotechnological applications as they enhance process efficiency and product yield [28]. Fractional factorial design and response surface methodology such as Plackett-Burman and Box-Behnken designs are prominent owing to their effectiveness in experimental design and process optimization [29, 30]. These approaches allow for the examination of how multiple factors interact with each other, which is often overlooked when studying variables individually [31].

Based on the above, the main objective of this study is to isolate a citrulline producing probiotic bacteria and optimize ADI and citrulline production using One factor at a time experiments followed by Plackett–Burman and Box–Behnken experimental designs.

## Materials and methods

### Sample collection

The samples were collected from the animal’s farm of College of Agriculture Assiut University, Assiut, Egypt. For safekeeping, the buffalo teats were wiped dry with sterile disposable towels, the first few milk streams were discarded to remove any potential contaminants. Then fresh milk samples were collected in a clean, sterilized polyethylene bags to the laboratory. Upon arrival, the samples were directly prepared for bacterial isolation.

### Isolation of bacteria from buffalo milk

Bacterial isolation was performed using 10-fold serial dilutions of the buffalo milk samples (pH = 7). A volume of 100 µL of the dilution was spread on sterilized nutrient agar medium (Himedia, India). The inoculated plates were incubated aerobically at 37 °C for 48 h. The growing colonies were enumerated, purified and preserved on Luria Bertani (LB) (Sigma-Aldrich, USA) slant agar at 4 ℃ for further use [32, 33].

### Molecular characterization of the isolated bacteria

The bacterial isolates were genetically identified using 16S rRNA gene sequencing after extraction of total genomic DNA [34, 35]. Overnight pre-growing bacterial cells were collected for DNA extraction. Patho-gene-spin DNA/RNA extraction kit provided by Intron Biotechnology Company; Korea was applied for bacterial DNA extraction. Amplification of a 1500 bp fragment within the 16S rRNA gene was achieved through PCR using two universal primers (Solgent Co., Ltd, Bio-Industry Development Site, 63 − 10 Hwaan-Dong, Yuseong-Gu, Daejeon, South Korea). The primers were 27F (5’-AGAGTTTGATCCTGGCTCAG-3’) and 1492R (5’-GGTTACCTTGTT ACGACTT-3’). For identification purposes, the isolate sequences were aligned and subjected to a BLAST search against the GenBank database of 16 S rRNA gene sequences www.ncbi.nlm.nih.gov/blast/Blast.cgi.

Using 16 S rRNA gene sequences obtained from GenBank, a phylogenetic tree was constructed with MEGAlign (version 5.05) software to determine the isolate’s taxonomic affiliation [36, 37].

### Screening for arginine deiminase (ADI) producer

A single pure colony was tested for ADI production on a screening medium consisting of 0.01% glucose. 1.5% L-arginine (Sigma-Aldrich, USA), 0.002% NaCl, 0.075% K_2_HPO_4_, 0.05% MgC1_2_, 0.01% MnCl_2_, 0.0005% FeSO_4_, 0.1 M CaCO_3_, 1.7% agar, pH 7.0 and 0.005% phenol red, as the pH indicator. The plates were incubated for 3–5 days at 40 °C. The colonies that were surrounded with pink color rings were selected as ADI producers [38].

### Screening of probiotic activity

#### Antimicrobial activity

The antimicrobial activity of the isolated bacteria was detected on six human pathogenic bacteria: *Escherichia coli*, *Klebsiella pneumonia*,* Serratia sp*,* Staphylococcus aureus*, methicillin-resistant *Staphylococcus aureus* (MRSA), *Streptococcus pneumoniae* were provided by Microbiology Lab, Assiut University educational hospital, Assiut, Egypt. The pathogenic bacteria were grown on nutrient broth for 18 h at 37 °C. Their turbidity was separately adjusted to OD600 = 0.3 and aseptically streaked onto Muller Hinton (MH) agar medium (Himedia, India). A 6 mm paper disc saturated with 20 µL of 24 h old of a potential probiotic bacterial isolate placed on the MH medium which was previously inoculated with pathogenic bacteria. The plates were incubated for 24 h at 37 °C. The antimicrobial activity was determined via formation of inhibition zone around the disc [38, 39].

#### Tolerance to gastric juice and intestinal juice consisting of bile salt

The resistance of isolated bacteria to artificial gastrointestinal juices was determined using the methods previously described by [40]. Artificial digestive fluids were prepared to simulate the stomach and intestinal environments.

- *Artificial gastric juice (AGJ)*: This solution consisted of 0.85% sterile saline solution containing 0.1% pepsin. The pH was adjusted to either 2.0 or 4.0 using 6 M hydrochloric acid (HCl).
- *Artificial intestinal juice (AIJ)*: it was prepared by adding either 0.3% or 0.5% bovine bile salt (Sigma-Aldrich, USA) to a buffer containing 12.4 g/L K_2_HPO_4_, 11.37 g/L trisodium citrate dehydrate, 7.6 g/L KH_2_PO_4_, and 6 g/L (NH_4_)_2_SO_4_. The pH of AIJ was adjusted to either 6.0 or 7.0.

Subsequently, 10% aliquot of a pre-grown bacterial culture at a density of 10^9^ CFU/mL was inoculated into either AGJ or AIJ and incubated at 37 °C for 2 h in a water bath [41].

#### Determination of the survival rate

Bacterial viability was tested by spreading 200 µL of a tested solution onto a nutrient agar plate and incubating at 37 °C for 24 h. The survival rate (SR) was calculated based upon the number of bacteria at zero and after 24h of incubation as follows:

1 $${\rm{SR }}\left( {\rm{\% }} \right){\rm{ = log}}\left( {{{{\rm{CFU/mL}}\,{\rm{after}}\,{\rm{24h}}} \over {{\rm{CFU/mL}}\,{\rm{at}}\,{\rm{0h}}}}} \right){\rm{ \times 100}}$$

#### Temperature and salinity tolerance

The temperature tolerance assay was performed by inoculating a pre-grown bacterial culture (10%) into nutrient broth (NB)-containing flasks, and each flask was incubated separately at 45, 50, 55, and 60 °C. For salinity tolerance assay, a pre-grown bacterial culture (10^8^ CFU/mL) was inoculated into NB supplemented with 2%, 4%, and 6% NaCl. After incubation for 24 h, The bacterial viability was determined, and SR was calculated as the method described before based upon Eq. (1).

#### Phenol sensitivity assay

The assay was performed to evaluate the survival rate of the bacteria in the presence of phenol solution. A pre-grown bacterial isolate (10^8^ CFU/mL) was inoculated in NB supplemented with phenol (0.4%) and incubated at 37 °C for 24 h. To determine the bacterial population, a 100 µL aliquot from a serial dilution was plated onto nutrient agar plates and incubated for 24 h. Bacterial counts were performed at both 0 h and 24 h [42].

#### Antibiotics sensitivity

Antibiotic sensitivity of the isolates was determined using the Kirby-Bauer disk diffusion method on MH agar. The overnight (24 h) broth cultures of isolates were swabbed uniformly on the surface of media using sterilized cotton swabs. Antibiotic discs (Cefotaxime, Rifampicin, Norfloxacin, Streptomycin, Erythromycin, and Ceftazidime) (Biomaster, Sweden) were placed on the media and incubated at 37 °C for 24–48 h. The zones of inhibition were also measured [39].

### Production of ADI from isolated bacteria

An overnight-growing ADI-producing bacterial culture (2% inoculum) was inoculated into minimal L-arginine medium (glucose 1%, yeast extract 0.5%, peptone 0.5%, L-arginine 1%, NH_4_Cl 0.15%, K_2_HPO_4_ 0.1%, MgSO_4_ · 7H_2_O 0.05%, MnSO_4_ · 4H_2_O 0.01%, NaCl 0.002%, pH 7.0). The flasks were incubated at 40 °C with shaking at 125 rpm for 3 days. Bacterial cells were collected and subjected to the ADI activity assay [43].

#### Preparation of intracellular enzyme extract

A volume of 5 mL of L-arginine-growing bacterial culture was centrifuged at 10,000 rpm for 5 min. The pellet was washed with phosphate buffered saline PBS (pH 7.0). The cells were resuspended in 100 mL sodium acetate buffer (0.2 M, pH 6.0) and ultrasonicated for 1 min (10 s pulses) via UltraTurax, T 2.5 at 4 ℃ to disrupt the cells. Then it was centrifuged for 10 min at 10,000 rpm and 4 ℃ to remove cell debris [44].

#### Determination of ADI activity

The ADI activity assay was performed according to the method described by [45]. The reaction mixture consisted of 500 µL of L-arginine dissolved in phosphate buffer (150 mM, pH 7.5) and 500 µL of the prepared intracellular enzyme extract. The enzymatic reaction was terminated after incubation at 40 °C for 30 min by the addition of 10% (w/v) tri citric acid. The mixture was centrifuged for 5 min at 10,000 rpm. The supernatant (50 µL) was mixed with 200 µL of Nessler’s reagent and the color developed was determined at 500 nm. One unit (U) of ADI was defined as the amount of enzyme required to release one µmol/min of ammonia under standard assay conditions [44, 46].

### Determination of accumulative L-citrulline in the medium

Diacetyl monoxime thiocarbazide assay was performed for photometric determination of the extracellular citrulline which accumulated due to ADI hydrolyzing activity on L-arginine [47]. The cell-free supernatant obtained from minimal L-arginine-grown bacteria was used.

- Reagent A consisted of 80 mM diacetyl monoxime and 2 mM thiosemicarbazide (Sigma-Aldrich, USA) in deionized water.
- Reagent B consisted of an acid mixture (H_3_PO_4_-H_2_SO_4_, 3:1 v/v) and 0.1 mM FeCl_3_ in a deionized water. A volume of 200 µL of the reaction mixture was added to 60 µL of the test sample mixture.

A volume of 50 mL of reagent A was mixed with 100 mL of reagent B. To determine the concentration of extracellular citrulline, a volume of 200 µL of reagents mixture was added to 60 µL of bacterial cell-free supernatant and boiled for 15 min at 100 ℃. After cooling, the absorbance of the product was detected at 530 nm using a UV-vis spectrophotometer [48]. The concentration of the produced L-citrulline in the reaction was estimated based on the citrulline (Sigma-Aldrich, USA) standard curve.

One unit (U) of ADI activity is defined as the amount of enzyme that hydrolyzes 1 micromole (µmol) of L-arginine into 1 µM of L-citrulline per minute under optimal assay conditions.

### Optimization of culture medium for L-citrulline production

#### Preliminary optimization using one-factor-at-a-time (OFAT) method

To assess the influence of different carbon and nitrogen sources on citrulline production by the isolated bacteria, a preliminary screening was conducted using the conventional OFAT method. The minimal L-arginine broth medium was modified by replacing its original carbon and nitrogen sources with individual supplements of various carbon sources (glucose, sucrose, or starch) and nitrogen sources (yeast extract, urea, or NH_4_NO_3_) [49]. Overnight pre-grown bacterial cells were provided as an inoculum. The impact of temperature was determined by incubation of media at 28, 35, 40 ℃ over a period 3–7 days. At the end of each experiment, the accumulative citrulline was determined using a method described before.

#### Statistical optimization via Plackett–burman design (PBD)

Eight variables were selected for this study: Incubation period (h), temperature (℃), pH, peptone, yeast, glucose, L-arginine and dipotassium monohydrogen phosphate K_2_HPO_4_ (Table 1). Fourteen experimental runs were conducted to analyze the influence of eight variables on citrulline production. Each variable was tested at two levels: a high level denoted by “+” and a low level denoted by “-” [50]. The high and low levels were chosen to be sufficiently distinct to ensure that any significant effect of the variable could be readily observed. Each run was performed in triplicate, and the reported results represent the average of these variables. This experimental design employed a first-order model to screen the variables, meaning it assumes that the effects of each variable on citrulline production are independent and additive. The equation of the first-order model is as follows:

2 $${\rm{Y = }}{{\rm{\beta }}_{\rm{0}}}{\rm{ + }}\sum {{{\rm{\beta }}_{\rm{i}}}\,{{\rm{X}}_{\rm{i}}}}$$

where Y was the response (ADI activity or citrulline production), β_0_ was the model intercept, and β_i_ was the estimated variable [51].

**Table 1:** Experimental variables and levels used in the PBD for optimal citrulline production by isolated BH-01

Factors	Name (unit)	Minimum low (− 1)	Maximum high (+ 1)
**A**	Temperature (℃)	30	40
**B**	pH	3	7
**C**	Incubation time (days)	3	7
**D**	L-arginine(g/L)	0.3	0.7
**E**	Peptone(g/L)	0.3	0.7
**F**	Yeast(g/L)	0.3	0.7
**G**	Glucose(g/L)	0.5	1.5
**H**	K_2_HPO_4_(g/L)	0.1	0.3

#### Statistical optimization via Box Behnken design

The conditions for citrulline production by isolated bacteria were optimized. Twenty-seven quadratic Box-Behnken runs were performed using four independent variables as follows: temperature (A), incubation time (B), L-arginine g/L (C), and peptone g/L (D). The summary of the applied variables with their ranges was illustrated in (Table S1). A statistical model was developed to investigate the relationships between the effective variables and their influence on both ADI activity and citrulline production [52]. The significance and accuracy of this model were evaluated using statistical analysis of variance (ANOVA) within Design Expert 13.0 statistical software (Stat-Ease Inc., Minneapolis, USA). A p-value of less than 0.05 was considered statistically significant, and the coefficient of determination (R²) was also analyzed to assess the model’s accuracy.

### Statistical analysis

For the probiotics activity and OFAT experiments, the mean standard deviations of the results from three independent experiments were calculated and analyzed using one-way ANOVA followed by grouping information using the Fisher LSD method and 95% confidence. Minitab statistics software version 19.0. P-values less than 0.05 were deemed to be statistically significant.

For statistical optimization designs, Design Expert 13.0 statistical software (Stat-Ease Inc., Minneapolis, USA) was used to analyze the experimental data using multiple regression analysis. The analysis focused on three types of relationships: linear, quadratic, and interaction effects. The importance of each effect was evaluated using Pareto analysis of variance (ANOVA). Additionally, an F-test was conducted to assess their statistical significance at a 5% level (*p* less than  0.05). Finally, to better understand the relationship between the response and the independent variables, response surface plots were generated based on the regression models. These plots visually represent how the response changes as the independent variables are adjusted.

## Results

### Isolation and molecular identification of ADI producing bacteria

A total of five aerobic ADI-producing bacterial isolates were isolated from a buffalo milk sample. These isolates were characterized by the formation of pink zones around their growing colonies on L-arginine agar plates. The isolated BH-01 was selected as the highest ADI producer due to the formation of the widest pink zone. The isolates were preserved in bacterial culture collection in the Assiut University Mycology Center with accession number AUMC B-498.

**Fig. 1:**
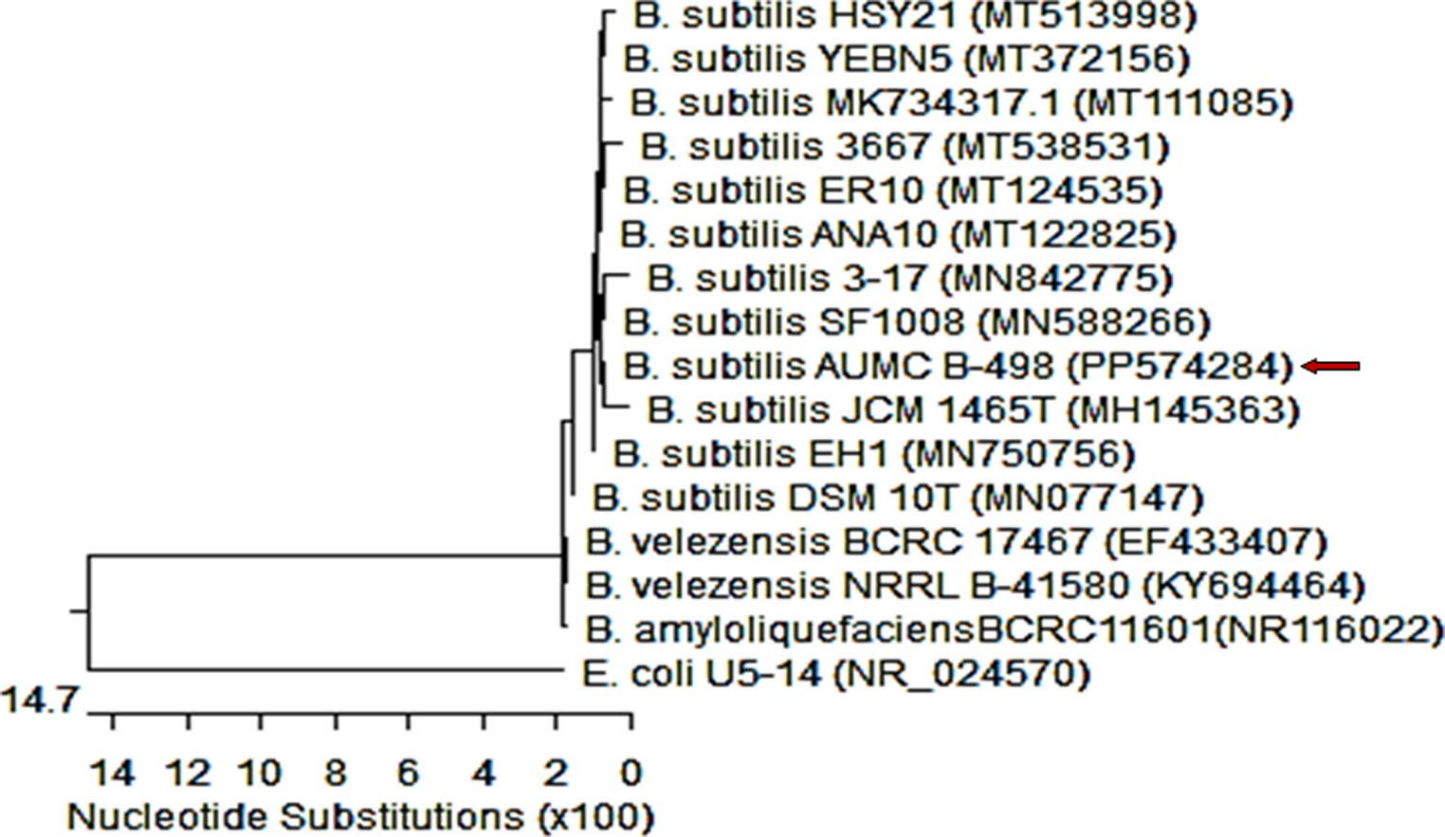
Phylogenetic tree based on 16 S rDNA gene sequencing of the bacterial strains *Bacillus subtilis* AUMC B-498, arrowed) aligned with closely related sequences of bacterial strains accessed from the GenBank. The two bacterial strains showed 99.86 − 100% identity and 99 − 100% coverage with several strains of *Bacillus subtilis* including the type of material *Bacillus subtilis* JCM1465 with GenBank accession no. (MH145363). *Escherichia coli* is included in the tree as an outgroup strain, *B* = *Bacillus*, *E* = *Escherichia*

The ADI-producing BH-01 was characterized through phylogenetic analysis based on 16 S rRNA gene sequence comparison. The alignment revealed a high similarity between the 16 S rRNA sequence of BH-01 and that of *Bacillus subtilis*. To further solidify BH-01’s phylogenetic position, a phylogenetic tree was constructed using 16 S rRNA sequences of various *Bacillus subtilis* species retrieved from the GenBank database. The tree revealed that the bacterial strain showed 99.86 − 100% identity and 99 − 100% coverage with several strains of *Bacillus subtilis*, including the strain *Bacillus subtilis* JCM1465 with GenBank accession no. (MH145363) as demonstrated in Fig. 1.

The 16 S rRNA gene nucleotide sequence of the isolated strain BH-01 has been deposited in the GenBank nucleotide sequence database under the accession number PP574248.1. The organism is *Bacillus subtilis* strain AUMC B-498.

### Probiotic activity of the isolated bacteria

**Fig. 2:**
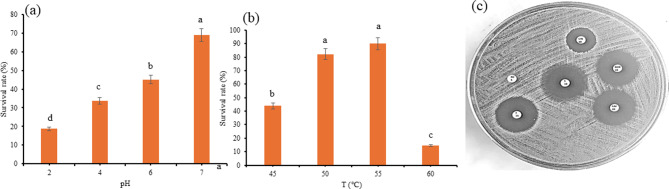
The impact of gastrointestinal pH (**a**), temperature (**b**) and antibiotics responses (**c**) on the survival rate of bacterial isolate BH-01. Plotted values are the means of triplicate treatments ± the standard deviation of the mean. The impact of the variable corresponding to each treatment and not sharing the same letters are significantly different according to the Fisher LSD Method and 95% Confidence

In the current study, the tested bacteria BH-01 exhibited resistance to low pH value. The isolate exhibited survival rates (25.4–40.6%) in the simulated gastric juice with pH values of 2.0 and 4.0, while it exhibited high survival rates (65.05–85.9%) in the simulated intestinal juice at pH 6.0 and 7.0, as illustrated in Fig. 2a. On the other hand, the isolate showed resistance to bile salt concentrations (0.3 and 0.5%) over 2 h of incubation, however it exhibited greater resistance at 0.3% bile salt with a survival rate of 70% after 2 h. The survival rate at 0.5% bile salt after 2 h significantly (*p* ≤ 0.01) decreased when compared with the survival rate at 0.3% Table 2. For phenol resistance, the isolate exhibited 60% survival rate at 0.4% of phenol solution Table 2.

Regarding the salinity impact, the results in Table 2. showed that high NaCl concentration significantly (*p* ≤ 0.01) suppressed BH-01 viability. At low salt concentrations (2%), the isolate showed the highest survival rate of 55.56%. On the other hand, the isolate BH-01 showed resistance to high temperature up to 60 ℃. The highest(*p* ≤ 0.01) survival rate (95.9%) was recorded at 45 ℃. High temperature 60 ℃ showed significant suppression of the bacteria growth with the survival rate (14.2%) Fig. 2b.

To evaluate the inhibitory effect of the isolated BH-01 on the growth of six pathogenic bacteria pathogens, the disc diffusion method was performed. Antagonistic activities against *E. coli*, *K. pneumonia*, *Serratia* sp., *S. aureus*, MRSA, and *S. pneumoniae* were detected. The potential probiotic BH-01 exhibited antipathogenic activity against all tested bacteria except *Serratia* sp. as illustrated in Table 3.

The result of antibiotic activity on the selected isolate BH-01 in Table 3; Fig. 2c displayed that the potential probiotic bacteria was sensitive to cefotaxime, norfloxacin, streptomycin, erythromycin, and Ceftazidime (the inhibition zone:10–15 mm) while it showed resistance to rifampicin (5 µ/disc) and no inhibition zone was observed.

**Table 2:** The impact of bile salt concentration, salinity, and phenol solution on the viability of BH-01

Treatment	Bile salt (%)				NaCl (%)			Phenol (%)
**Concentration**	0.3		0.5		2	4	6	0.4
	1 h	3 h	1 h	3 h				
**Survival rate (%)**	61.2 ± 1.2	70 ± 2.1	50.56 ± 0.4	40.5 ± 1.2	55 ± 0.7	30 ± 1.3	10 ± 0.2	60 ± 1.4

±: The values are the means standard deviations of triplicate measurements

**Table 3:** The antagonistic activity of the potential probiotic BH-01 against some pathogenic bacteria and the antibiotics impact on the potential probiotic BH-01

Pathogen	*K. pneumonia*	*Serratia sp*	*S. aureus*	MRSA	*S. pneumoniae*	*E.coli*
	Zone of inhibition (mm)					
**BH-01 culture**	6.2 ± 0.2	0	6.1 ± 0.2	8.1 ± 0.5	4.2 ± 0.1	7.6 ± 0.4
**Antibiotic**	**Cefotoxime** (30 µ/disc)	**Rifampicin** (5 µ/disc)	**Norfloxacin** (10 µ/disc)	**Streptomycin** (10 µ/disc)	**Erythromycin** (35 µ/disc)	**Ceftazidime** (30 µ/disc)
	**Zone of inhibition (mm)**					
	10 ± 1.2	R	11.2 ± 0.2	15 ± 0.7	11 ± 0.2	13 ± 0.3

±: The values are means standard deviations of triplicate measurements R: resistant

#### OFAT optimization

**Fig. 3:**
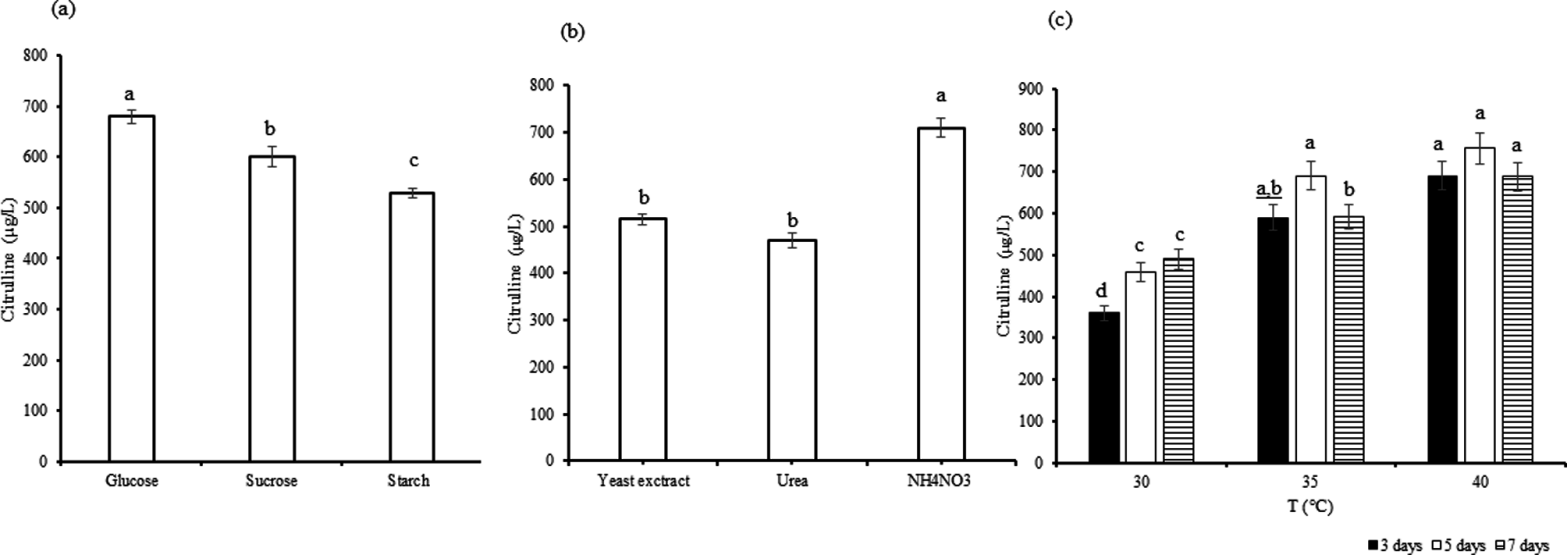
The OFAT optimization parameters, impact of carbon source (**a**), nitrogen source (**b**) and incubation period and temperature (**c**) on L-citrulline production by isolate BH-01. Plotted values are the means of triplicate treatments ± the standard deviation of the mean. The impact of the variable corresponding to each treatment that are not sharing the same letters are significantly different according to the Fisher LSD Method and 95% Confidence

The conventional OFAT experiment was performed to investigate the key environmental requirements such as carbon, nitrogen and temperature and incubation periods which impact on citrulline production via potential probiotic BH-01. Regarding the studied carbon sources, glucose showed significantly (*p* ≤ 0.05) higher L-citrulline production (680 ± 1.8 µg/L) whereas NH_4_NO_3_ was the optimum nitrogen source with the optimum L-citrulline production (710.3 ± 0.46 µg/L) (Fig. 3a, b). The results in Fig. 3c depicted the effect of temperature and incubation period of the production of citrulline. It was noticed that the production of citrulline significantly (*p* ≤ 0.05) increased by elevating temperature, and it reached a maximum of 710.3 ± 0.46 µg/L at 40 ℃. While the incubation period did not significantly affect L-citrulline production (*p* ≤ 0.05), the highest yields of 460.3, 690.5, and 756.6 µg/L were observed at 5 days for 30, 35, and 40 ℃, respectively.

#### Analysis of Plackett–Burman results to identify the effective variables

The environmental fermentation factors (pH, temperature, and incubation time) and nutritional fermentation factors (L-arginine, glucose, peptone, yeast extract, and K_2_HPO_4_ concentration) were tested as influential variables. These variables were selected based upon the OFAT results. They were assessed for their impact on both ADI activity and the concentration of accumulated citrulline. The results in Table S2 showed the average values obtained for the fourteen experiments of the Plackett–Burman design. The Plackett-Burman design was employed to generate regression equations that identified the factors significantly influencing the two responses. The equations were expressed by the coefficient of R^2^, which was 0.99 and 0.98 for ADI activity and L-citrulline concentration, respectively. Therefore, the model equations for both responses can be represented as:

3 $$\eqalign{{\rm{ADI}}\,{\rm{activity}}\,\left( {{\rm{U/mL}}} \right){\rm{ }} & {\rm{ = 0}}{\rm{.8066 + 0}}{\rm{.0669A + 0}}{\rm{.0032B}} \cr & {\rm{ - 0}}{\rm{.1009C + 0}}{\rm{.1506D + 0}}{\rm{.0594E}} \cr & {\rm{ - 0}}{\rm{.1511F - 0}}{\rm{.1399G - 0}}{\rm{.0628H}} \cr & {\rm{ - 0}}{\rm{.1214AB + 0}}{\rm{.0641AC - 0}}{\rm{.0517AD}} \cr}$$

4 $$\eqalign{{\rm{Citrulline}}\,{\rm{concentration}}\,\left( {{\rm{\mu g/L}}} \right) & {\rm{ = 420}}{\rm{.79 + 40}}{\rm{.63A - 11}}{\rm{.46B}} \cr & {\rm{ - 46}}{\rm{.06C - 46}}{\rm{.06D + 34}}{\rm{.96E}} \cr & {\rm{ - 68}}{\rm{.57F - 73}}{\rm{.41G28}}{\rm{.04H}} \cr & {\rm{ - 58}}{\rm{.11AB + 24}}{\rm{.38AC}} \cr}$$

The coefficient R^2^ values obtained confirmed the effectiveness of the design in predicting the influence of the variables on both ADI activity and citrulline production by the potential probiotic BH-01. The p-value less than 0.05 indicated that the model terms were significant as illustrated in Table 4. For ADI activity model, A, C, D, E, F, G and H were significant model factors while A, C, F and G were significant factors for citrulline production model. It was noticed that B factor was non-significant for both tested models. The adeq Precision ratio of 23.65 and 12.49 for ADI activity and citrulline production. They were greater than 4 indicates an adequate signal and the model was fit.

**Table 4:** Regression coefficients and their statistical significance for variables using ANOVA for Plackett-Burman Design. Significant coefficients at *P* < 0.05 level

Source	Sum of Squares	df	Mean Square	F-value	*p*-value	Sum of Squares	df	Mean Square	F-value	*p*-value
Response	ADI(U/mL)					Citrulline(µg/L)				
Model	0.5059	11	0.0460	52.84	0.0187	1.723E + 05	10	17225.80	15.29	0.0230
A-Temperature	0.0569	1	0.0569	65.38	0.0150	21133.41	1	21133.41	18.76	0.0227
B-pH	0.0001	1	0.0001	0.0686	0.8179	1276.20	1	1276.20	1.13	0.3653
C-Incubation period	0.0611	1	0.0611	70.23	0.0139	20624.58	1	20624.58	18.30	0.0235
D-L-arginine	0.1114	1	0.1114	128.00	0.0077	36944.24	1	36944.24	32.79	0.0106
E-Peptone	0.0212	1	0.0212	24.36	0.0387	7335.11	1	7335.11	6.51	0.0838
F-Yeast Extract	0.0725	1	0.0725	83.28	0.0118	28210.27	1	28210.27	25.04	0.0154
G-Glucose	0.1321	1	0.1321	151.74	0.0065	44980.94	1	44980.94	39.92	0.0080
H-K_2_HPO_4_	0.0266	1	0.0266	30.54	0.0312	6563.53	1	6563.53	5.83	0.0947
AB	0.0442	1	0.0442	50.82	0.0191	12527.71	1	12527.71	11.12	0.0446
AC	0.0123	1	0.0123	14.15	0.0640	2205.05	1	2205.05	1.96	0.2563
AD	0.0080	1	0.0080	9.22	0.0935					
Pure Error	0.0017	2	0.0009			602.31	2	301.15		
Cor Total	0.5076	13				1.756E + 05	13			
C.V.%=3.75						C.V.% = 8.19				
R^2^ = 0.99						R^2^ = 0.98				
adjusted R^2^ = 0.9778						adjusted R^2^ = 0.94				
AdeqPrecision = 23.65						AdeqPrecision12.4989				

For ADI: and while for Citrulline production. SS: sum of squares, df: degree of freedom, MS: mean square C.V.: coefficient of variation

**Fig. 4:**
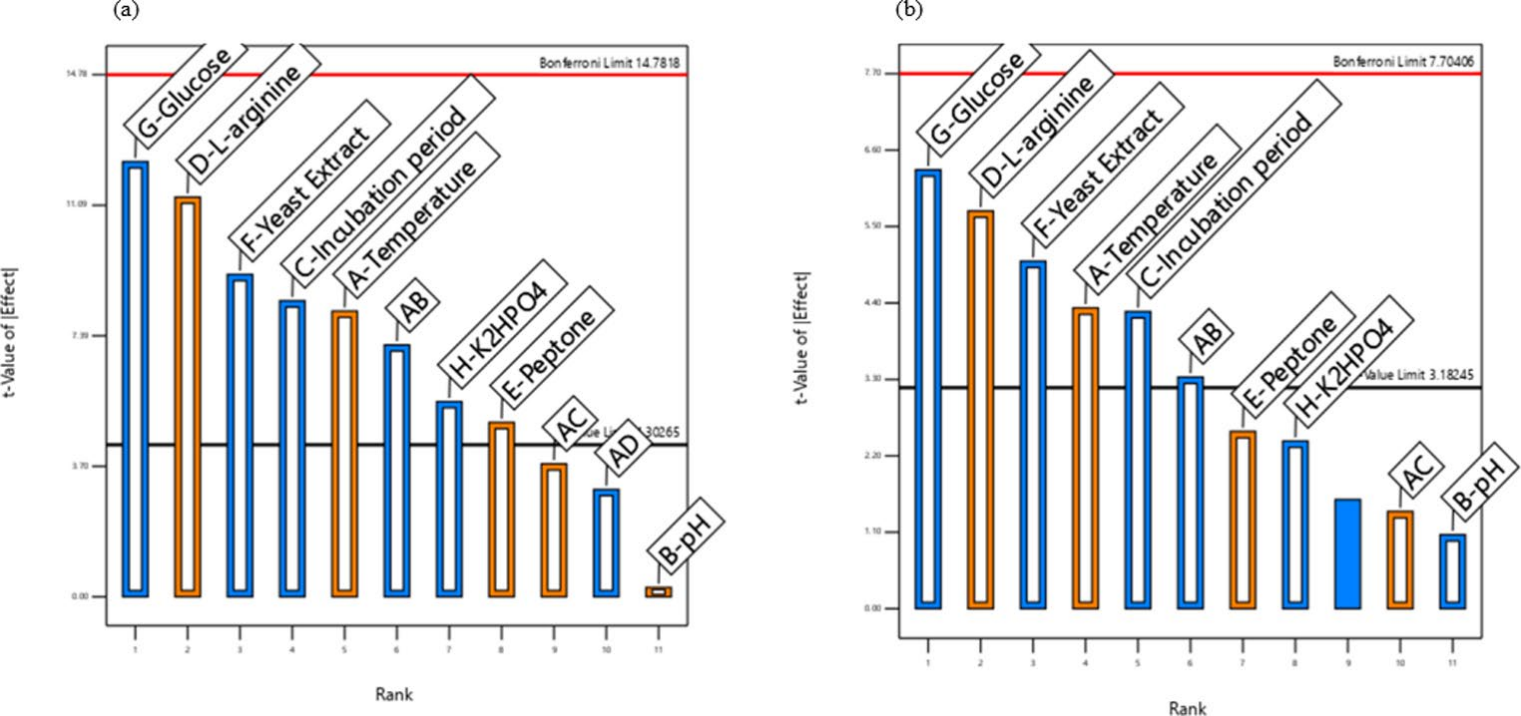
Pareto charts for Plackett–Burman screening, depicting the frequency distribution of the data presented for ADI activity (**a**) and L-citrulline production (**b**). Black line indicates the significant variables at a 0.05 level. Blue and orange column indicated the positive and negative influences of variable on the tested responses, respectively

The Placket–Burman design experiments were then analyzed using Pareto charts (Fig. 4a and b). The results revealed that L-arginine, temperature, and peptone concentration displayed significant (*p* ≤ 0.05) positive correlations on the tested responses (ADI activity and citrulline production). On the other hand, glucose, yeast extract, and incubation period displayed significant (*p* ≤ 0.05) negative correlations on the tested responses. Furthermore, validation of the statistical analysis was displayed in Figure S1, which showed the relation between the actual and predicted data for ADI activity and L-citrulline production, illustrating good reproduction of the experimental data in all cases.

It was observed that L-arginine, temperature, and incubation period were effective factors in the process. However, peptone concentration was an effective factor only during the process of ADI production. Therefore, L-arginine concentration, temperature, incubation period and peptone were selected as the relevant parameters for the second stage of the experiment.

#### Analysis of effective factors via Box Behnken design (BBD)

Following the initial screening with the Plackett-Burman design, the most impactful factors influencing ADI activity and L-citrulline production by BH-01 were selected for the next step of optimization using the Box-Behnken design. This approach provided comprehensive insights into the key factors affecting both responses and their optimal operating conditions. Therefore, temperature (A), incubation period (B), L-arginine (c) and peptone were selected as important factors influencing ADI activity and citrulline production by *B.subtilis* BH-01. The Box–Behnken matrix and the experimental results are listed in Table S2. The optimization study aimed to maximize both ADI activity (U/mL) and citrulline yield (µg/L). To model the relationships between these responses and the independent variables, two second-order polynomial equations were employed as follows:

5 $$\eqalign{{\rm{ADI}}\,{\rm{activity}}\,\left( {{\rm{U/mL}}} \right) & {\rm{ = + 1}}{\rm{.14 + 0}}{\rm{.1592A - 0}}{\rm{.0586B}} \cr & {\rm{ + 0}}{\rm{.1178C + 0}}{\rm{.0307D}} \cr & {\rm{ - 0}}{\rm{.1817A - 0}}{\rm{.0881B - 0}}{\rm{.0314 C}} \cr}$$

6 $$\eqalign{{\rm{Citrulline}}\,{\rm{concentration}}\,\left( {{\rm{\mu g/L}}} \right) & {\rm{ = + 632}}{\rm{.45 + 93}}{\rm{.05A - 38}}{\rm{.37B}} \cr & {\rm{ + 77}}{\rm{.37C + 20}}{\rm{.05D - 22}}{\rm{.87A}} \cr & {\rm{ - 20}}{\rm{.461B - 17}}{\rm{.09C}} \cr}$$

ANOVA was used as a statistical method to analyze the data and compare the variation within groups. The F-values (7.3 for ADI activity and 12.12 for citrulline production) and their corresponding p-values (both < 0.01) indicate that the regression models are not statistically significant at the 0.01 level. However, the high coefficient of determination (R^2^) values (0.80 and 0.88 for the respective models) suggests a good fit of the models to the data. Additionally, the agreement between adjusted R^2^ and predicted R^2^ values further supports this conclusion. While the lack-of-fit p-value for the ADI activity model (0.05) was not significant compared to the pure error, it’s important to note that both models might benefit from further investigation or potential refinement.

**Fig. 5:**
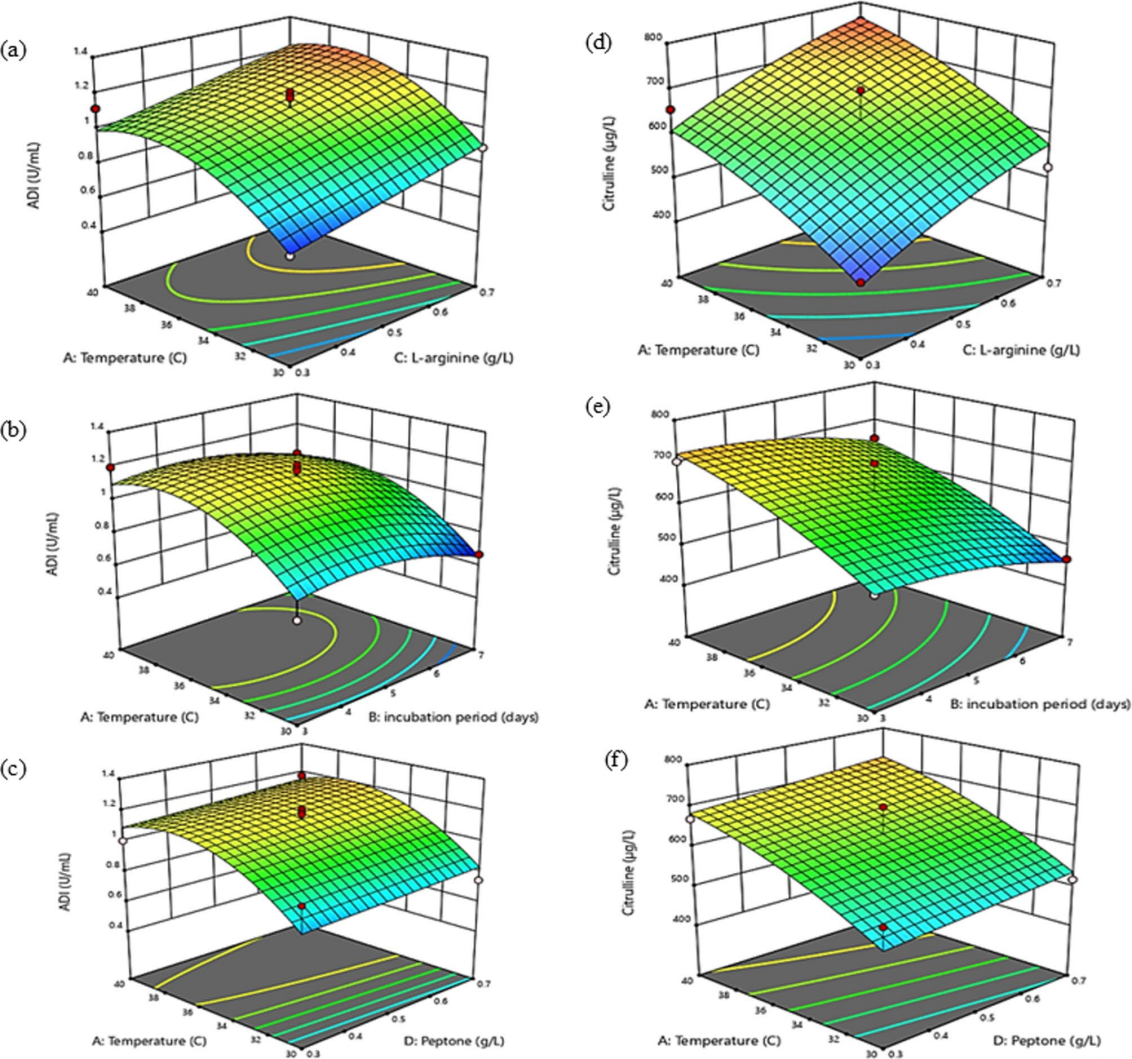
Three-dimensional response surface plots showing the effect of temperature, L-arginine, incubation periods and peptone on ADI (**a**, **b** and **c**) and L-citrulline production (**d**, **e** and **f**)

After determination of the main variables, three-dimensional surface response plots were generated to determine their significance (Fig. 5a, b, c and d. 5e and 5f). Based upon ANOVA analysis results, it was noticed that the impact of L-arginine concentration and temperature on ADI activity as well as citrulline production were significant (*p* < 0.05). Therefore, ADI activity as well as citrulline production exhibited escalating trends with increase in both L-arginine concentration and temperature (Fig. 5a, b and c). The analysis revealed no significant interaction between incubation period and peptone concentration on ADI activity, neither in their linear nor quadratic terms. However, the temperature exhibited a significant negative effect on ADI activity, particularly in its quadratic term. Additionally, incubation period had a significant positive linear effect on citrulline production, but its quadratic term showed a non-significant negative effect (Fig. 5d, e and f) and (Table 5). The optimization revealed that the maximum citrulline production was 632.5 µg/L and the arginine deiminase (ADI) activity was 1.42U/mL at 0.5 g/L of L-arginine as a substrate, 0.5 g/L of peptone at 35 ℃ and 5 days of incubation.

**Table 5:** Regression coefficients and their statistical significance for variables using ANOVA for Box-Behnken design. Significance coefficients at *P* < 0.05 level

Source	SS	df	MS	F-value	*p*-value	SS	df	MS	F-value	*p*-value
ADI(U/mL)						**Citrulline(µg/L)**				
Model	0.7362	7	0.1052	7.36	0.0002	2.032E + 05	7	29031.69	21.15	< 0.0001
A-Temperature	0.3040	1	0.3040	21.28	0.0002	1.039E + 05	1	1.039E + 05	75.70	< 0.0001
B-incubation period	0.0412	1	0.0412	2.88	0.1057	17666.02	1	17666.02	12.87	0.0020
C-L-arginine	0.1664	1	0.1664	11.65	0.0029	71833.90	1	71833.90	52.34	< 0.0001
D-Peptone	0.0113	1	0.0113	0.7898	0.3853	4824.03	1	4824.03	3.52	0.0763
A²	0.1981	1	0.1981	13.87	0.0014	3136.98	1	3136.98	2.29	0.1470
B²	0.0466	1	0.0466	3.26	0.0869	2512.69	1	2512.69	1.83	0.1919
C²	0.0059	1	0.0059	0.4127	0.5283	1751.95	1	1751.95	1.28	0.2726
Residual	0.2715	19	0.0143			26075.36	19	1372.39		
Lack of Fit	0.2709	17	0.0159	54.71	0.0181	18653.37	17	1097.26	0.2957	0.9420
Pure Error	0.0006	2	0.0003			7422.00	2	3711.00		
Cor Total	1.01	26				2.293E + 05	26			
C.V.%=11.2						C.V.%= 6.12				
R^2^ = 0.80						R^2^ = 0.88,				
adjusted R^2^ = 0.778						adjusted R^2^ = 0.85				
predicted R^2^ = 0.69						predicted R^2^ = 0.80				

SS: sum of squares, df: degree of freedom, MS: mean square and C.V.: coefficient of variation

## Discussion

L-citrulline is a non-essential amino acid and has been utilized as an oral supplement [53]. It exhibits several beneficial effects on gastrointestinal surgery, including reducing inflammation in the intestines, supporting the rebuilding of the intestinal lining after major surgery, and preventing muscle loss in malnourished individuals [4, 54]. Citrulline is mainly produced via bacteria through arginine deiminase (ADI) pathway, which is composed arginine deiminase Therefore, in this study, the arginine deiminase and L-citrulline producing *Bacillus subtilis *(BH-01) was isolated from raw buffalo milk sample. It was reported that many isolated *Bacillus subtilis* exhibited arginine degradation and induction of arginine deiminase [55], arginase [56].

The bile salt, phenol solution and acidic pH resistance, viability at high temperature and antagonistic activity on pathogenic bacteria confirmed that the BH-01 strain has probiotic characteristics [57, 58]. This finding is in accordance with [59], which mentioned that most of the probiotic and health-promoting bacteria were isolated from milk and dairy products. Because the species of family Bacillaceae can be produced as extremely durable, thick, proteinaceous coated endospores, they are particularly well-suited for probiotic applications. These spores enable the bacteria to survive in mammalian GI tract conditions such as high temperature, low pH, high concentration of bile salts [60]. phenol is not a major or intended product of digestion, however it can be a minor metabolic byproduct depending on the host diet and the composition of gut microbiota. Therefore, phenol survivability assay is a valuable test in screening potential probiotic strains. It assesses their ability to survive in the harsh acidic environment of the stomach, a key characteristic for effective probiotics [61].

Environmental and nutritional factors showed significant impact on the production of L-citrulline and activity of ADI [62, 63]. The optimum production of L-citrulline was estimated when glucose was utilized as a caron source, which was in agreed with the report of [62]. The optimum nitrogen source was NH_4_NO_3_, this may be attributed to the presence of ammonia in the media that promoted the accumulation of citrulline during ADI pathway [38]. Temperature regulated the bacteria ADI pathway as the accumulation of citrulline by BH-01 increased by increasing the temperature, which is in accordance with the results obtained by [62]. They mentioned that L-citrulline content produced from *Pediococcus acidilactici* and *Weissella confusa* reduced to less than half at 15 ℃. Furthermore [16], displayed that enzymatic production of L-citrulline via ADI pathway was induced at 30 and 37 ℃. Based uopon these results, L-citrulline yield from BH-01 is a promising candidate in the biotechnological applications. This may be attributed to it toleranance to broad pH and temperature ranges due to presence of endospores, and it could utilize low-cost substrates such as peptone for production. Conversely, the production of citrulline by *E. coli* [13], *Corynebacterium glutamicum* [14], *Lactobacillus* sp [4]. and *Pseudomonas* sp [64], which may have safety concerns, limited or slow production yield and need refined substrates.

To optimize the enzyme production and accumulative products manufacturing costs, screening the most important components and optimizing the growing conditions are crucial. Plackett–Burman and Box–Behnken designs are powerful statistical tools in biotechnology. Their combined application facilitates the systematic optimization of processes, leading to improved efficiency and product yields. Plackett–Burman designs are widely used for screening medium components and process parameters to identify those that significantly influence production [65]. The results of the Plackett–Burman design experiments provided insights into the optimal and significant parameters which negatively or positively influence production process [65]. The key variables that influenced citrulline production were temperature, L-arginine concertation, pH, incubation time, peptone, yeast extract and K_2_HPO_4_. Neutral pH optimizes ADI enzymatic activity, as the enzyme typically functions best under these conditions as recently described by [66]. The specified temperature aligns with the mesophilic growth range of *Bacillus subtilis* and supports enzyme stability [56]. L-Arginine supplementation is considered the primary substrate and inducer for the ADI pathway [67]. It was previuosly mentioned that yeast extract and peptone supplementation improved *Bacillus subtilis* growth and metabolic activity by providing essential nutrient, cofactors and they serving as protective agents [67]. For industrial processes, these variables may improve consistency and robustness of the fermentation process, ensuring reproducible citrulline yields with less cost [13].

The Box-Behnken design is a valuable tool within response surface methodology, particularly suited for simultaneously optimizing multiple parameters while minimizing the required number of experimental runs. BBD helps determine the ideal combination of variables like pH, temperature, and substrate concentrations (e.g., glucose, L-arginine, peptone, and yeast extract) to enhance BH-01 growth and metabolic activity as described by [68]. The information it offers regarding curvature, optimal factor levels, and regions of maximum or minimum response is of great significance as it aids in optimizing processes, enhancing quality, and allocating resources efficiently [65, 69]. Besides, BBD reduces waste by identifying the minimal net sufficient concentrations of nutrients equired for optimal production, ensuring efficient resource utilization and minimizing inhibitory effects of excessive substrate concentrations [52].

Among the analyzed variables, the L-arginine concentration and temperature emerged as crucial factors in the citrulline production and ADI activity. The accumulation of L-citrulline is attributed to the metabolism of L-arginine by bacteria ADI pathway [26]. This may be attributed to L-arginine being actively transported into the BH-01 cells via a specific arginine transport system, such as the arginine-ornithine antiporter [70]. In addition to the presence of L-arginine in the medium acts as an inducer and upregulates the expression of genes in the ADI pathway [70]. Therefore, by increasing its concentration in the media, it lead to increase the ADI activity and L-citrulline production [71]. Regarding to the temperature, maintaining production temperatures within the mesophilic range (30–35 ℃) ensures enzyme stability and efficient metabolic processes in *Bacillus subtilis*. Deviations from this range can lead to reduced enzyme activity and compromised bacterial growth [56].

The industrial production of citrulline via microbial ADI activity provides many implications such as (1) cancer therapy [6, 10, 72], (2) it is used for improving exercise performance, cardiovascular health, and reducing muscle fatigue [73–75]. (3) it can be used as feed additives in species with inefficient arginine metabolism to enhance livestock health and growth rates [76]. Moreover, the optimization of citrulline production via ADI pathways in microbial systems reduces production costs and increases yield [13, 77]. ADI and citrulline production pathways can be engineered into microbial systems to create efficient biosensors or biocatalysts for specific industrial reactions [13]. Therefore, the citrulline production using potential probiotic BH-01 offers unique advantages in safety, cost-effectiveness, and consumer appeal, making it a competitive choice compared to other bacterial systems. Its dual functionality as a citrulline producer and probiotic adds value in food, nutraceutical, and pharmaceutical applications.

In conclusion, the isolated bacteria were identified as *Bacillus subtilis*. It could survive at acidic pH, high bile salt concentration, high temperature, inhibit human pathogenic bacteria. Statistical analyses were performed with Plackett–Burman discarding the effect of the other variables on the ADI activity and citrulline production. From the insights obtained by the Plackett–Burman analysis, the study conducted the Box–Behnken design to investigate the optimum responses achievable with the four most influential variables.

## Electronic supplementary material

Below is the link to the electronic supplementary material.

Supplementary Material 1

## Data Availability

Sequence data that support the findings of this study have been deposited in the NCBI with the primary accession code PP574248.1. Other data were provided in the supplementary file.
