# Supplementary material for: Optimization of citrulline production from a Bacillus subtilis BH-01 isolated from raw buffalo milk
Source: BMC Microbiol. 2025 Feb 10;25:71. doi: 10.1186/s12866-025-03768-0 (PMC11809042; doi:10.1186/s12866-025-03768-0)
Supplement: Supplementary file 1 — Supplementary Material 1 [file 12866_2025_3768_MOESM1_ESM.docx]

**Supplementary materials**

**Journal Name: BMC Microbiology**

**Title**

**Optimization of Citrulline production from a *Bacillus subtilis* BH-01 Isolated from Raw Buffalo Milk**

**Marwa A.K. Mansour^1^, Salah Ali^1^, Manal A. M. Hassan^3^, Fify A. Gabra^4^, Asmaa M. M. Mawad^2*^**

**^1^** Botany and Microbiology Department, Faculty of Science, Al-Azhar University, 71524, Assiut, Egypt**.**

**^2^** Botany and Microbiology Department, Faculty of Science, Assiut University, 71516, Assiut, Egypt**.**

**^3^**Food and technology Department, Faculty of Agriculture, Assiut University, 71526, Assiut, Egypt**.**

**^4^** Faculty of Medicine, Assiut University, 71526, Assiut, Egypt**.**

***Corresponding author: Asmaa M. M. Mawad.** [**a.mawad@aun.edu.eg**](mailto:a.mawad@aun.edu.eg)**,** **Mobile Number: +966582500619**

**Table 1S**: Box–Behnken experimental design with coded perational variables (A: temperature (℃),B: incubation period(days), C: L-arginine (g/L) and D: peptone (g/L)).

| Run | A | B | C | D |  | ADI (U/mL) | Citrulline(µg/mL) |
| --- | --- | --- | --- | --- | --- | --- | --- |
| 1 | -1 | 0 | 1 | 0 |  | 0.8939 | 525.824 |
| 2 | 1 | -1 | 0 | 0 |  | 1.19449 | 702.641 |
| 3 | 0 | 1 | 0 | -1 |  | 0.9329 | 548.765 |
| 4 | 0 | 0 | 1 | 1 |  | 1.31562 | 773.897 |
| 5 | -1 | 0 | 0 | -1 |  | 0.93652 | 550.894 |
| 6 | 0 | -1 | 1 | 0 |  | 1.17362 | 690.364 |
| 7 | 0 | -1 | 0 | 1 |  | 1.17451 | 690.89 |
| 8 | 0 | -1 | 0 | -1 |  | 1.0909 | 641.706 |
| 9 | 0 | 0 | 0 | 0 |  | 1.18791 | 698.771 |
| 10 | 0 | -1 | -1 | 0 |  | 0.956231 | 562.489 |
| 11 | 1 | 0 | -1 | 0 |  | 1.1149 | 655.824 |
| 12 | 0 | 0 | -1 | -1 |  | 0.781966 | 459.98 |
| 13 | 0 | 1 | -1 | 0 |  | 0.834013 | 490.596 |
| 14 | 0 | 0 | 1 | -1 |  | 1.1823 | 695.47 |
| 15 | 0 | 0 | 0 | 0 |  | 1.17417 | 593.258 |
| 16 | -1 | -1 | 0 | 0 |  | 0.663833 | 534.134 |
| 17 | 1 | 0 | 0 | 1 |  | 1.174 | 704.899 |
| 18 | 0 | 0 | -1 | 1 |  | 1.07143 | 542.024 |
| 19 | 0 | 0 | 0 | 0 |  | 1.2081 | 593.258 |
| 20 | 1 | 0 | 1 | 0 |  | 0.984266 | 756.133 |
| 21 | 1 | 0 | 0 | -1 |  | 1.00399 | 668.817 |
| 22 | 1 | 1 | 0 | 0 |  | 1.0109 | 652.382 |
| 23 | 0 | 1 | 1 | 0 |  | 1.28588 | 628.05 |
| 24 | -1 | 0 | 0 | 1 |  | 0.744416 | 517.699 |
| 25 | -1 | 1 | 0 | 0 |  | 0.669916 | 465.182 |
| 26 | -1 | 0 | -1 | 0 |  | 0.663831 | 430.383 |
| 27 | 0 | 1 | 0 | 1 |  | 0.816627 | 576.823 |

**Table2S**: Effect of variables on ADI activity and citrulline production produced by BH-using Plackett-Burman Design.

| **Run**  **Order** | **A** | **B** | **C** | **D** | **E** | **F** | **G** | **H** | **ADI(U/mL)** | **Citrulline(µg/L)** | | |
| --- | --- | --- | --- | --- | --- | --- | --- | --- | --- | --- | --- | --- |
| **1** | 30 | 5 | 3 | 0.7 | 0.7 | 0.7 | 0.5 | 0.3 | 0.968 | | 517 |  |
| **2** | 40 | 9 | 3 | 0.7 | 0.3 | 0.3 | 0.5 | 0.3 | 1.060 | | 570.98 |  |
| **3** | 30 | 5 | 3 | 0.3 | 0.3 | 0.3 | 0.5 | 0.1 | 0.872 | | 460.5294 |  |
| **4** | 40 | 9 | 3 | 0.7 | 0.7 | 0.3 | 1.5 | 0.1 | 1.024 | | 550.1789 |  |
| **5** | 40 | 9 | 7 | 0.3 | 0.7 | 0.7 | 0.5 | 0.3 | 0.605 | | 303.4706 |  |
| **6** | 30 | 9 | 3 | 0.3 | 0.3 | 0.7 | 1.5 | 0.3 | 0.414 | | 190.98 |  |
| **7** | 40 | 5 | 3 | 0.3 | 0.7 | 0.7 | 1.5 | 0.1 | 0.761 | | 395.2353 |  |
| **8** | 40 | 5 | 7 | 0.7 | 0.3 | 0.7 | 0.5 | 0.1 | 1.046 | | 562.87 |  |
| **9** | 40 | 5 | 7 | 0.3 | 0.3 | 0.3 | 1.5 | 0.3 | 0.745 | | 385.8235 |  |
| **10** | 30 | 9 | 7 | 0.3 | 0.7 | 0.3 | 0.5 | 0.1 | 0.91 | | 482.8824 |  |
| **11** | 30 | 9 | 7 | 0.7 | 0.3 | 0.7 | 1.5 | 0.1 | 0.614 | | 308.7647 |  |
| **12** | 30 | 5 | 7 | 0.7 | 0.7 | 0.3 | 1.5 | 0.3 | 0.69 | | 353.4706 |  |
| **13** | 30 | 5 | 7 | 0.7 | 0.7 | 0.3 | 1.5 | 0.3 | 0.631 | | 318.7647 |  |
| **14** | 30 | 5 | 7 | 0.7 | 0.7 | 0.3 | 1.5 | 0.3 | 0.66 | | 335.8235 |  |


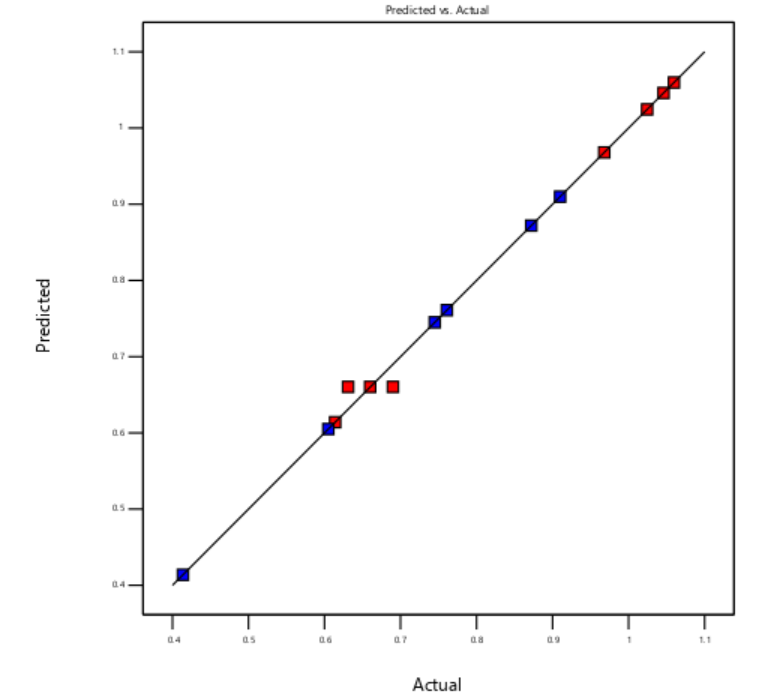

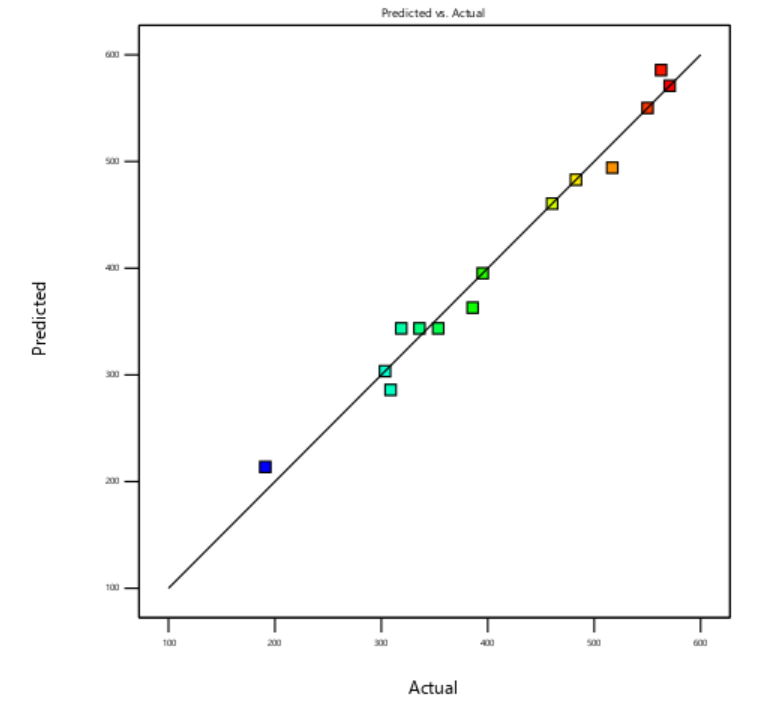


**(a)**

**(b)**

**Fig.1S**: Experimental actual and predicted plots of ADI activity (a) and citrulline production (b) by the fitted models
